# Systematic review of patient reported quality of life following stereotactic ablative radiotherapy for primary and metastatic liver cancer

**DOI:** 10.1186/s13014-017-0818-8

**Published:** 2017-06-29

**Authors:** Adam Mutsaers, Jeffrey Greenspoon, Cindy Walker-Dilks, Anand Swaminath

**Affiliations:** 1 Department of Medicine, McMaster University, 1280 Main Street West, Hamilton, ON L8S 4 L8 Canada; 2 Juravinski Cancer Centre at Hamilton Health Sciences, Hamilton, ON Canada; 3 Department of Oncology, McMaster University, Juravinski Cancer Centre, 699 Concession St, Hamilton, ON L8V 5C2 Canada; 4 Program in Evidence Based Care, McMaster University, Juravinski Hospital, 60 (G) Wing, 2nd Floor, 711 Concession Street, Hamilton, ON L8V 1C3 Canada

**Keywords:** SABR, SBRT, Stereotactic, Hepatocellular carcinoma, Liver metastases, Systematic review, Quality of life, QOL

## Abstract

**Background:**

Stereotactic ablative radiotherapy (SABR) is a safe and effective modality in patients with liver cancer who are ineligible for other local therapies. However SABR is not current standard of practice and requires further validation. Patient reported quality of life (QOL) is key to this validation, yet no systematic reviews to date have been performed to analyse QOL following liver SABR. QOL is a critical part of therapy evaluation, particularly in disease states with short life expectancy. The purpose of this study was to conduct a systematic review of QOL outcomes for liver SABR.

**Materials and methods:**

MEDLINE and EMBASE databases from 1996 to October 2015 were queried to obtain English language studies analysing QOL following liver SABR. Included studies described patient-reported QOL as either a primary or secondary endpoint, and analysed QOL change over time. Studies were screened, and relevant data were abstracted and analysed.

**Results:**

Of 2181 initially screened studies, 5 met all inclusion criteria. Extracted studies included a total of 392 eligible patients with hepatocellular carcinoma, liver metastases and intrahepatic cholangiocarcinoma. Four studies were prospective in design, and only one study was a conference abstract. Extracted studies were heterogeneous in dose prescription used (11–70 Gy in 3–30 fractions), in addition to reported QOL metrics (EORTC QLQ C-15 PAL,/C-30/LM-21, EuroQol 5D, FACT-Hep, FLIC) and final endpoints (range 6 weeks to 12 months). Despite this there were few statistically significant declines in QOL scores following SABR. Four studies demonstrated transient fatigue in the first 1–4 weeks, while 2 studies showed transient worsening of appetite at 1 month. In all but one instance (loss of appetite at 6 weeks), levels returned to insignificant difference baseline by the final endpoints. All studies showed no significant QOL decline in any domain at their respective endpoints. In studies with overlapping QOL tools, estimates of 3-month post SABR global QOL were similar.

**Conclusion:**

Results of this systematic review demonstrate well-preserved post liver SABR QOL. These findings strengthen the argument for liver SABR, and should aim to support future comparative effectiveness trials with other local modalities including surgery, chemoembolization and radiofrequency ablation, with a focus on QOL outcomes as an important endpoint.

**Electronic supplementary material:**

The online version of this article (doi:10.1186/s13014-017-0818-8) contains supplementary material, which is available to authorized users.

## Background

Primary and metastatic liver tumours are among the most common tumours for both men and women globally [1]. Furthermore, these tumours can cause significant mortality and morbidity with symptoms including pain, vomiting, fatigue, nausea and fever [2]. Over the last decade improvements in radiation treatment planning and delivery have renewed interest in and enabled the use of advanced radiotherapeutic modalities for treating primary and metastatic liver cancers. Stereotactic ablative radiotherapy (SABR) is an emerging modality in patients with liver cancer who are ineligible for other local therapies including surgical resection, transplant, radio-frequency ablation (RFA) and transarterial chemoembolization (TACE) primarily due to co-morbidities or advanced disease [3, 4].

SABR involves the highly conformal and image guided delivery of hypofractionated external beam radiotherapy. Typically it involves delivering high, ablative doses of radiation over a shorter period of time than conventional fractionated radiotherapy [5]. SABR has been used for both inoperable primary liver cancers including hepatocellular carcinomas (HCC) and intrahepatic cholangiocarcinoma (IHC), as well liver metastases (LM) from various primaries [6]. SABR has been shown to be effective with respect to local control with rates at 1 year ranging from 71 to 100%, and at 2 years between 64% and 92% [7–15]. It has also been shown to be reasonably safe, with limited grade 3 toxicities across a number of trials, particularly with respect to overall liver function, and luminal gastrointestinal toxicity [16].

However SABR for liver cancer is not current standard of practice despite its potential promise. In order to validate the increased offering of this promising therapy, objective systematic data regarding impact on quality of life (QOL) is required. There is some emerging evidence around patient quality of life (QOL) metrics in liver SABR based on single institution data. Analyzing QOL metrics are a critical part of therapy evaluation for several reasons including: QOL being predictive of clinical prognosis; providing effective comparisons with current standards of practice; and enabling greater patient and physician understanding of risk-reward tradeoffs in clinical decision making [17]. This is particularly true in disease states with short life expectancy. No systematic reviews to date have been performed to analyse and summarize the evidence on QOL for primary or metastatic liver cancers. The purpose of this study was to synthesize the evidence and determine if QOL across studies is well preserved following liver SABR.

## Materials and methods

### Search strategy

MEDLINE and EMBASE databases from 1996 to October 2015 were queried to obtain English language studies analysing QOL following SABR for liver cancers. A Health Research Methodologist (CWD) assisted in development of the search strategy and completed the search. One additional study was included via a grey literature search after reviewing pertinent studies. Details regarding the search strategy and strings can be found in Additional file 1: Appendix A.

### Inclusion criteria

Study review from the primary search was initially conducted independently by 3 screeners (AM, AS, JG), and with discrepancies resolved by consensus. Included studies relevant for the review met the following criteria:

1. Liver as primary or metastatic site of radiotherapy
2. SABR as primary modality of radiotherapy
3. Study type was either a) randomized controlled trial (RCT), b) meta-analyses of RCTs, prospective studies, or retrospective studies (n > 10).
4. Patient-reported QOL was specified as either a primary or secondary endpoint
5. QOL change over time was analyzed
6. Studies included greater than 10 patients
7. Studies were published in English

### Data abstraction

Conference abstracts and journal articles were both included in the final abstraction, and if relevant and feasible, primary authors for these studies were contacted to collect further data beyond the published abstract(s). Included studies were extracted by two reviewers (AM & AS) while relevant data were abstracted and analysed by a single reviewer (AM).

## Results

### Search results

**Fig. 1:**
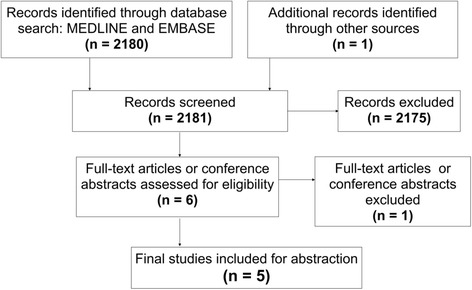
PRISMA Flow Diagram

A PRISMA 2009 Flow Diagram in Fig. 1 summarizes the review process. Of 2181 initially screened studies, 4 met all inclusion criteria (Klein et al., Shun et al., Mendez Romero et al., Thibault et al.) and were analysed, with one additional study having met criteria after being identified from a grey literature search (Law et al.) [18–22].

## Study characteristics

Included study characteristics are summarized in Table 1. Extracted study dates ranged from 2008 to 2015, included a total of 388 eligible patients, and 4/5 studies were prospective in design with the exception being Law et al. Four of the extracted studies were final publications, with one conference abstract (Thibault et al.) also abstracted. In this case, the primary author was contacted and no further information beyond the original abstract was available. Individual studies included patients with HCC, LM and IHC with varying weights. Extracted studies were heterogeneous in dose prescription used (11–70 Gy in 3 – 30 fractions), as well as in QOL evaluation tools (European Organisation for Research and Treatment of Cancer (EORTC) QLQ C-15 PAL,/C-30/LM-21, EuroQol 5D, FACT-Hep, FLIC), pre-treatments with other therapies, disease staging and final endpoints (range of 6 weeks to 12 months) analyzed. QOL follow up methods and venues were mixed. Questionnaires were completed by patients in their home in 1 study (Mendez Romero et al.), were done either in clinic or at home, via telephone or self administered in 1 (Klein et al.) and were not explicitly outlined in 3 studies (Law et al., Thibault et al., Shun et al.

**Table 1:** Summary of study design

Investigator	Year	Location	Pop. Characteristics	Publication type	Study Design	N	Median Age	Treatment technique	QOL tool	Assessment Timeline
Thibault [21]	2014	Canada	Mets (30)	Conference Abstract	Prospective Longitudinal Cohort	30	65 (40–88)	SBRT 30-60Gy, 3-6#	QLQ-C15PAL QLQ-LM21	Baseline, 1, 6, 12 weeks post treatment
Shun [22]	2008	Taiwan	HCC (99)	Journal article	Prospective Longitudinal Cohort	99	62.42 (12.6)	SRT - Mean dose 42.6Gy	FLIC-QOL	1 week pre treatment, weekly x6 weeks during
Mendez Romero [20]	2008	Netherlands	Mets (19) HCC (9)	Journal article	Prospective Longitudinal Cohort	25	68 (37–81)	SBRT 3x12.5Gy 5x5Gy 3x10Gy	EQ-5D EQ-5D VAS QLQ C-30	1 month pre + 1, 3, 6 months post treatment
Klein [18]	2015	Canada	Mets (86) HCC (98) IHC (21)	Journal article	Prospective Longitudinal Cohort	205	67 (30–90)	Image guided SBRT 24–60 GY/6#	QLQ-C30 FACT-Hep	Baseline and 1,3,6,12 months post treatment
Law [19]	2012	Hong Kong	HCC (33)	Journal article	Prospective Longitudinal Cohort	33	69 (47–89)	Highly-conformal RT 55GY/10#	FACT-Hep	Baseline, 3, 6 months

### QOL measurement tools

The FACT-Hep tool contains 45 questions, scaled 0–4, covering the past 7 days over 2 major categories of well-being: General, which includes 27 questions surrounding physical, emotional, social and family functioning, and site specific which includes 18 Hepatobiliary related symptom questions including abdominal pain, swelling, and diarrhea [23]. EORTC has several validated QOL tools used in these studies (QLQ C-15 PAL,/C-30/LM-21). Versions differ by number of questions, and specificity for LM patients. The QLQ-C30 contains 30 questions, scaled 1–4, covering the past week surrounding emotional, physical, role, social, and cognitive function as well as specific symptoms including nausea, pain, and fatigue, and finally overall health and QOL [24]. In a recent literature review by Ghandi et al. on the topic of QOL tools in HCC patients, found that the QLQ-C30 and FACT-Hep have been used most commonly of over 70 available oncologic QOL tools. Both have been validated as primary outcome measures, and are used readily used to assess secondary endpoints [25].

To our knowledge, no thorough comparisons between QOL tools in liver cancers has been published. Klein et al. compared two scales and suggests that the QLQ-C30 may be more sensitive, while the FACT-Hep better at differentiating between initial diagnosis. More research is required.

### QOL results across studies

Summarized findings from the extracted studies are found in Table 2. There were few clinically or statistically significant declines in QOL scores, or variables comprising the indices following SABR, at any time-points. With respect to variables comprising the QOL scales, three studies demonstrated increased fatigue transiently in the first 1–4 weeks, while one study (Shun et al.) showed sustained increase in fatigue at 6 weeks (final end point). Two studies (Klein et al., Shun et al.) showed statistically significant worsening of appetite at 1 month, while in Klein et al. the metric returned to an insignificant difference from baseline by three months. All studies showed no significant decline in mean QOL at their respective final endpoints; in fact both Mendez Romero et al. and Shun et al. showed statistically insignificant improvement in mean QOL scores at respective endpoints (6 months, *p* = 0.69 and 6 weeks, *p* = 0.75 respectively).

**Table 2:** QOL Results Post SABR to Liver

Investigator	QOL Outcome Studied	Significant Findings
Thibault [21]	Mean QOL at 1 week, 6 weeks, 3 months, by component	~ No clinically or statistically significant decline in QOL at final endpoint (3 months) in either QOL tool ~ Mostly stable QOL item scores (12/15 in QLQ-C15, 21/21 in QLQ-LM21) in measurements over 12 weeks ~ Increased fatigue, decreased global health status at 1 week post treatment on C15 (*p* = 0.049, *p* = 0.033); both returned to baseline at 6 weeks and 3 months
Shun [22]	Mean QOL at 1 week pretreatment, weekly for 6 weeks during	~ Non-clinically and non-statistically significant increase in QOL scores at 6 weeks ~ Mean symptom severity (measured on Symptom Severity Scale) increased at final endpoint ~ Mean depression (measured on POMS-D Depression subscale) increased at final endpoint ~ Functional status (*p* = 0.003), depression (*p* = 0.0001), symptom severity significantly (*p* = 0.0002), level of albumin (*p* = 0.001) associated with changes in QOL ~ Radiation dosage not significant factor in QOL during treatment
Mendez Romero [20]	~Mean QOL at pretreatment and 1/3/6 month ~Symptom and functional domains	~ No statistically significant decline in mean QOL over 6 months ~ Fatigue at 1 month showed significant decline when compared baseline (*p* = 0.004); returned to baseline thereafter; was only functional or symptom-specific domain to show significant decline; though trends showed worsening symptoms across time points ~ Non statistically significant trend towards improvement noted at final endpoint ~ Baseline QOL of patients was lower than general population comparison group (*p* < 0.001)
Klein [18]	~ Mean QOL @ 0 1,3,6,12 months ~ Percent patients where QOL “improved” vs “Stable” vs “Worsened”), (MID = 10 on QLQ C30, vs 14 on FACT-Hep)	~ Beyond 3 months, no significant worsening vs baseline in both tools ~ Using FACT-Hep/QLQ-C30, 54%/48% reported stable QOL, 27%/39% showed clinically significant worsening, 19%/23% showed significant improvement at 12 months post treatment ~ Significant worsening from baseline in appetite (11.7 points) and fatigue (11.0 points) at 1 month, both return to baseline by 3 months on QLQ-C30 ~ Other variables show no clinically or statistically significant change from baselines ~ Higher baseline QOL predicts improved survival in both FACT-HEP (*p* = 0.001) and QLQ-C30 (*p* = 0.012) ~ LM patients experienced statistically significantly, but not clinically significant, better quality of life at 6 months when compared to HCC (*p* = 0.04); no difference between LM and IHC, or IHC and HCC patients
Law [19]	Mean QOL @ baseline, post treatment, 3, 6 months ~ MID > 5% change	~ No statistically or clinically significant change in FACT-Hep score in all time points vs baseline, trend towards poorer QOL at final end point (*p* = 0.09)

Shun et al. showed that symptoms of depression (measured by POMS depression subscale) (*p* = 0.0001), functional status (measured by single item ECOG-PS) (*p* = 0.0003), overall symptom severity (*p* = 0.0001), and albumin levels (*p* = 0.001) were all significantly predictive for changes in QOL scores during treatment. Further, when controlling for depressive status, functional status and serum albumin variables including lack of appetite (*p* = 0.0001), fatigue (*p* = 0.003) and nausea (*p* = 0.002) were found to be predictive of QOL over the course of therapy. Despite increases in average symptom severity of all 7 measured symptoms during the 6 weeks, overall quality of life remaining stable (113.8 at T = 0, 114.48 on FLIC scale) throughout treatment.

### Comparison of QOL using similar endpoints and evaluation tools

When analysing comparable endpoints and tools between studies, there are several findings. Klein et al. and Law et al. both used the FACT-Hep tool and demonstrated similar estimates of 3-month post SABR global QOL. Both demonstrated that QOL was unchanged from baseline at this time-point. Three studies (Klein et al., Thibault et al., Mendez Romero et al.) used the EORTC QLQ based (QLQ C-30 vs QLQ C-15 vs QLQ LM21) QOL evaluation tool and had scheduled follow up at one month. Klein et al. showed significant decreases in fatigue and appetite QOL scores at 1 month, while Mendez Romero et al. also showed significant worsening of fatigue (but not other QOL scores). In contrast, Thibault et al. showed universal symptom return to baseline at 1 month.

Klein et al. was the only study to examine difference in QOL changes by initial diagnosis (HCC and IHC versus LM). Analysis showed statistically favourable but not clinically significant mean QOL benefit for LM patients at 1(*p* = 0.003) and 6(*p* = 0.014) months using FACT-Hep when compared with HCC and IHC patients together, or HCC patients alone. Differing QOL score components between diagnoses were not reported.

Two studies (Mendez Romero et al., Shun et al.) compared baseline QOL to the general population of non-cancer patients in their respective geographies and demonstrated significantly lower baseline QOL in the patient population than the general population across all tools used. Furthermore, Shun et al. showed that baseline QOL was worse than a similar cohort with metastatic colon cancer being treated with chemotherapy.

## Discussion

Overall, SABR appears to be well tolerated from the patients’ perspective for both liver primaries and metastases across nearly all QOL categories beyond the acute phase. There appear to be few changes in QOL present in the acute phase, though evidence on the topic is currently limited, and studies varied in the tools used, and time-points analyzed.

Given the small number of studies, and studies that analysed multiple diagnoses collectively, it was not feasible to review QOL for liver metastases and primaries independently. There are inherent differences between these conditions that could affect baseline QOL including physiologic liver function and location and magnitude of other primary disease. All studies analysed QOL in a pre/post treatment longitudinal manner. As such, the change in score stemming from treatment, rather than the absolute level was most relevant outcome, and was shown to be preserved in all studies, regardless of primary diagnosis.

The findings in two studies of lower baseline QOL in cancer patients than in the non-cancer populations is not surprising. However, Shun et al. found that several factors including depressive mood, functional status, and symptom severity were significantly associated with QOL changes. These predictive variables may represent important intervention points for patients undergoing SABR. Supporting nutritional status, mental health and managing symptoms during and following treatment could improve QOL. Given that the most common changes in QOL across all studies were fatigue and nutrition status (appetite/nausea) over the first month following treatment, these symptoms should also be monitored to predict for changes in QOL, with symptom management interventions offered early or in a preventative manner, especially if the SABR dose distribution is limited by GI luminal tissues.

Currently there is insufficient head to head evidence comparing SABR QOL outcomes with those of more currently accepted therapies such as RFA, TACE or surgery. Therefore there is the potential for a significant research opportunity to explore randomized trials of SABR with QOL as an important primary or secondary endpoint. One such trial, RTOG 1112, is a phase III RCT evaluating the role of sorafenib with or without upfront SABR in patients with advanced HCC [26] QOL is being evaluated in this study and should provide further prospective evidence as to the comparative impact following liver SABR (with the hypothesis that at 6 months QOL should be no different in those patients receiving SABR followed by sorafenib as compared to sorafenib alone). A recently performed cost-effectiveness study of SABR versus RFA for colorectal liver metastases demonstrates that utilities are similar for both toxicity and treatment of recurrence for both modalities. However this is largely based on retrospective and small prospective/pooled analyses [27]. In order to determine if there is a clear benefit in both effectiveness without compromise of patient reported QOL, RCTs between SBRT and other local modalities including RFA need to be performed. Unfortunately such a trial (RAS study) was closed due to poor accrual [28].

Nonetheless, on the basis of this systematic review, there is some evidence to suggest that SABR can preserve short term QOL. Langenhoff et al. demonstrated, using similar evaluation tools (EQ-5D, EQ-5D VAS and QLQ C-30) that in a control group of inoperable patients with colorectal liver metastases, receiving no treatment had significantly lower QOL at 6 months. The surgical groups had increases in symptom severity transiently, but returned to baseline by 3 to 6 month [29, 30].

Reviews of QOL in other local therapies have produced varied results, with some studies suggesting stable (Rees et al., Toro et al.) or worse (Eid et al., Huang et al.) scores in patients undergoing liver resections for primary or metastatic disease [31–34]. Similar variations are seen post chemo/radio-embolization (Salem et al., Smits et al.) [35, 36]. Based on our analysis and limited comparable data, SABR is comparable or even a favourable alternative to other surgical and non surgical therapies from a QOL standpoint.

As an important adjunct to QOL, safety and toxicity are important factors to consider when evaluating emerging treatments. Of the studies reporting significant toxicities, Mendez Romero et al. disclosed one ‘potentially treatment related death’. Beyond this, patients who progressed or underwent other adjuvant therapies were excluded from the analysis to avoid confounders to QOL from these factors. Other studies did not exclude patients who underwent further therapy or progressed, including those who had an evolving disease course in the longitudinal QOL measurement. Law et al. demonstrated grade 3 toxicity (leukopenia, elevated liver enzyme and bilirubin) in 5/33 cases (15%), with no grade 4 toxicity. One patient died from cirrhosis during the study. Shun et al. revealed that hemoglobin and albumin decreased while ALT levels rose during the course of treatment, but did not comment on specific toxicity in individual patients. The authors concluded that this was an ‘imperceptible’ side effect of the treatment. The remaining two studies did not comment on toxicity within their respective populations, but analysed symptom changes along a number of dimensions (nausea, fatigue, etc.). Several other studies have demonstrated limited toxicities in SABR treatment to the liver, demonstrating the practice to be reasonably safe when planning and dose constraints are followed [7–15].

Several of the studies only published mean results of global QOL metrics. While an important summary measure, there may be population subsets that do better or worse than the average following SABR. For example, Klein et al. demonstrated diversity in outcomes with a minority of patients either improving or deteriorating to significant degrees vs baseline. A clinically significant improvement was seen in 19%/23% of patients at 1 year (FACT-Hep/QLQ-C30), while 27%/39% showed clinically significant worsening over this same time frame. It would be of value to consider such proportional differences, and larger studies with more granular reporting would be needed to evaluate drivers of these different responses.

One limitation of this analysis is that there is bias due to a lack of consensus on appropriate QOL measurement tools across studies, particularly in the post SABR setting. This makes it challenging to interpret findings in a coordinated way, and therefore perform a meta-analysis or pooled analysis of the data. A greater alignment with QOL evaluation tools for liver SABR (in comparison with other ablative therapies) will better support future comparisons between modalities. Other factors including the effects of radiation dose to liver and luminal GI organs, number of fractions, or target volume size may also represent potential drivers of QOL, and would be important in future study designs using similar evaluation tools.

Other issues preventing a larger meta-analysis were the varying endpoints of each study; 4/5 studies were at 6 months or less, including two which were 3 months or less. One study (Shun et al.) was done during active therapy. The tendency for side effects to subside in the months following therapy could explain the worsened symptoms demonstrated in the short term relative to endpoints of the other studies. Long term QOL post SABR is also less clear in this context, and in general, given the short follow-up of many trials. However, trending of all relevant metrics seem to indicate no significant decline directionally with increasing time from treatment. The review was comprised of 5 studies with 392 total patients representing a small sample size, in conjunction with varied dose fractionation, tumour size, patient comorbidities, baseline liver function and primary diagnoses across the studies. QOL completion in studies ranged between 36% and 85% percent (at 12 months and 6 weeks of follow-up respectively), and universally declined over time. No reported data was subsequently obtained from patients who did not respond. Missing data in QOL assessments are always an issue, which may be magnified when trying to pool data across multiple studies.

Ultimately, trials randomizing between SABR and other modalities will provide important data on QOL as well as local control, survival benefits and toxicities. Further research could seek to improve quality of data collection and reporting in several ways. Consistent QOL reporting pre-, during and following treatment in both the short and long term (1 year and beyond) could provide a clearer picture of the impact of treatment. Reporting on sub populations including those with different diagnoses (HCC versus metastases), and comparing those who improved and those who worsened could also provide insight towards QOL determinants. Additionally, consistent reporting of symptom or function related subscales will provide better understanding of QOL change drivers. Standardized QOL tool use and follow-up intervals would enable data-pooling for more significant analyses.

## Conclusions

Results of this systematic review demonstrate well-preserved post SABR QOL in patients with otherwise untreatable liver cancer, despite heterogeneity amongst the individual studies themselves. These findings merit further research to increase data collection, to validate and standardize QOL tools specific to SABR for liver cancers and their sub-populations, and to support comparative effectiveness trials of SABR with other local modalities in liver cancer including surgery, TACE and RFA, with a focus on QOL outcomes as an important endpoint. Continued research in this field will enable better discussions and shared decision making between patients and physicians with respect to complex treatment choices moving forward.

## Additional file

Additional file 1: Appendix A. Detailed search strategy. (DOCX 24 kb)

## Abbreviations

AM: Adam Mutsaers (author)
AS: Anand Swaminath (author)
CWD: Cindy Walker-Dilks (author)
HCC: Hepatocellular carcinoma
IHC: Intrahepatic cholangiocarcinoma
JG: Jeffrey Greenspoon (author)
LM: Liver metastases
QOL: Quality of life
RCT: Randomized Control Trial
RFA: Radio-frequency ablation
SABR: Stereotactic ablative body radiation
TACE: Transarterial chemoembolization

## References

[1] Bosch F, Ribes J, Díaz M, Cléries R (2004). Primary liver cancer: worldwide incidence and trends. Gastroenterology.

[2] Bydder S, Spry N, Christie D, Roos D, Burmeister B, Krawitz H, Davis S, Joseph D, Poulsen M, Berry M (2003). A prospective trial of short-fractionation radiotherapy for the palliation of liver metastases. Australas Radiol.

[3] Alongi F, Arcangeli S, Filippi A, Ricardi U, Scorsetti M (2012). Review and uses of stereotactic body radiation therapy for oligometastases. Oncologist.

[4] Tree A, Khoo V, Eeles R, Ahmed M, Dearnaley D, Hawkins M, Huddart R, Nutting C, Ostler P, van As N (2013). Stereotactic body radiotherapy for oligometastases. Lancet Oncology.

[5] Sahgal A, Roberge D, Schellenberg D, Purdie T, Swaminath A, Pantarotto J, Filion E, Gabos Z, Butler J, Letourneau D, Masucci G, Mulroy L, Bezjak A, Dawson L, Parliament M (2012). The Canadian association of radiation oncology scope of practice guidelines for lung, liver and spine stereotactic body radiotherapy. Clin Oncol.

[6] Boda-Heggemann J, Dinter D, Weiss C, Frauenfeld A, Siebenlist K, Attenberger U, Ottstadt M, Schneider F, Hofheinz R, Wenz F, Lohr F (2012). Hypofractionated image-guided breath-hold SABR (Stereotactic Ablative Body Radiotherapy) of liver metastases – clinical results. Radiat Oncol.

[7] Hoyer M, Roed H, Traberg Hansen A, Ohlhuis L, Petersen J, Nellemann H, Kiil Berthelsen A, Grau C, Aage Engelholm S, Von Der Maase H (2006). Phase II study on stereotactic body radiotherapy of colorectal metastases. Acta Oncol.

[8] Méndez Romero A, Wunderink W, Hussain S, De Pooter J, Heijmen B, Nowak P, Nuyttens J, Brandwijk R, Verhoef C, Ijzermans J, Levendag P (2006). Stereotactic body radiation therapy for primary and metastatic liver tumors: A single institution phase i-ii study. Acta Oncol.

[9] Herfarth K, Debus J, Wannenmacher M. Stereotactic Radiation Therapy of Liver Metastases: Update of the Initial Phase-I/II Trial. Controversies in Gastrointestinal Tumor Therapy. 2004;38:100–05. 10.1159/000078271 15458194

[10] Lee M, Kim J, Dinniwell R, Brierley J, Lockwood G, Wong R, Cummings B, Ringash J, Tse R, Knox J, Dawson L (2009). Phase I study of individualized stereotactic body radiotherapy of liver metastases. J Clin Oncol.

[11] Rusthoven K, Kavanagh B, Cardenes H, Stieber V, Burri S, Feigenberg S, Chidel M, Pugh T, Franklin W, Kane M, Gaspar L, Schefter T (2009). Multi-institutional phase I/II trial of stereotactic body radiation therapy for liver metastases. J Clin Oncol.

[12] Ambrosino G, Polistina F, Costantin G, Francescon P, Guglielmi R, Zanco P (2009). Image-guided robotic stereotactic radiosurgery for unresectable liver metastases: preliminary results. Anticancer Res.

[13] Goodman K, Wiegner E, Maturen K, Zhang Z, Mo Q, Yang G, Gibbs I, Fisher G, Koong A (2010). Dose-escalation study of single-fraction stereotactic body radiotherapy for liver malignancies. Int J Radiat Oncol Biol Phys.

[14] Scorsetti M, Comito T, Tozzi A, Navarria P, Fogliata A, Clerici E, Mancosu P, Reggiori G, Rimassa L, Torzilli G, Tomatis S, Santoro A, Cozzi L (2014). Final results of a phase II trial for stereotactic body radiation therapy for patients with inoperable liver metastases from colorectal cancer. J Cancer Res Clin Oncol.

[15] Rule W, Timmerman R, Tong L, Abdulrahman R, Meyer J, Boike T, Schwarz R, Weatherall P, Chinsoo Cho L (2010). Phase I dose-escalation study of stereotactic body radiotherapy in patients with hepatic metastases. Ann Surg Oncol.

[16] Pan C, Kavanagh B, Dawson L, Li X, Das S, Miften M, Ten Haken R (2010). Radiation-associated liver injury. Int J Radiat Oncol Biol Phys.

[17] Osoba D, Bezjak A, Brundage M, Pater J (2007). Evaluating health-related quality of life in cancer clinical trials: the national cancer institute of Canada clinical trials group experience. Value Health.

[18] Klein J, Dawson L, Jiang H, Kim J, Dinniwell R, Brierley J, Wong R, Lockwood G, Ringash J (2014). Prospective longitudinal assessment of quality of life for liver cancer patients treated with stereotactic body radiation therapy. Int J Radiat Oncol Biol Phys.

[19] Law A, Ng W, Lee M, Chan A, Fung K, Li F, Lao W, Lee A (2012). Treatment of primary liver cancer using highly-conformal radiotherapy with kv-image guidance and respiratory control. Radiother Oncol.

[20] Méndez Romero A, Wunderink W, van Os R, Nowak P, Heijmen B, Nuyttens J, Brandwijk R, Verhoef C, IJzermans J, Levendag P (2008). Quality of life after stereotactic body radiation therapy for primary and metastatic liver tumors. Int J Radiat Oncol Biol Phys.

[21] Thibault I, Chu W, Chan K, Erler D, Chow E, Chung H (2014). Quality of life in patients treated with stereotactic ablative body radiation therapy (SABR) for liver metastases. Int J Radiat Oncol Biol Phys.

[22] Shun S, Chiou J, Lai Y, Yu P, Wei L, Tsai J, Kao C, Hsiao Y (2008). Changes in quality of life and its related factors in liver cancer patients receiving stereotactic radiation therapy. Support Care Cancer.

[23] Heffernan N, Cella D, Webster K, Odom L, Martone M, Passik S, Bookbinder M, Fong Y, Jarnagin W, Blumgart L (2002). Measuring health-related quality of life in patients with hepatobiliary cancers: the functional assessment of cancer therapy–hepatobiliary questionnaire. J Clin Oncol.

[24] EORTC. (n.d.). Retrieved March 8, 2017, from http://groups.eortc.be/qol/glossary

[25] Ghandi S, Khubchandani S, Iyer R (2014). Quality of life and hepatocellular carcinoma. J Gastrointest Oncol.

[26] Dawson, L. A. (n.d.). RTOG | Clinical Trials | Study Number 1112. Retrieved September 21, 2016, from https://www.rtog.org/ClinicalTrials/ProtocolTable/StudyDetails.aspx?study = 1112

[27] Kim H, Gill B, Beriwal S, Huq M, Roberts M, Smith K (2016). Cost-effectiveness analysis of stereotactic body radiation therapy compared with radiofrequency ablation for inoperable colorectal liver metastases. Int J Radiat Oncol Biol Phys.

[28] Radiofrequency Ablation Versus Stereotactic Radiotherapy in Colorectal Liver Metastases - ClinicalTrials.gov [https://clinicaltrials.gov/ct2/show/record/NCT01233544]

[29] Langenhoff B, Krabbe P, Peerenboom L, Wobbes T, Ruers T (2006). Quality of life after surgical treatment of colorectal liver metastases. Br J Surg.

[30] Wietzke-Braun P, Schindler C, Raddatz D, Braun F, Armbrust T, Nolte W, Ramadori G (2004). Quality of life and outcome of ultrasound-guided laser interstitial thermo-therapy for non-resectable liver metastases of colorectal cancer. Eur J Gastroenterol Hepatol.

[31] Rees J, Blazeby J, Brookes S, John T, Welsh F, Rees M (2014). Patient-reported outcomes in long-term survivors of metastatic colorectal cancer needing liver resection. Br J Surg.

[32] Toro A, Pulvirenti E, Palermo F, Di Carlo I (2012). Health-related quality of life in patients with hepatocellular carcinoma after hepatic resection, transcatheter arterial chemoembolization, radiofrequency ablation or no treatment. Surg Oncol.

[33] Eid S, Stromberg A, Ames S, Ellis S, McMasters K, Martin R (2006). Assessment of symptom experience in patients undergoing hepatic resection or ablation. Cancer.

[34] Huang G, Chen X, Lau W, Shen F, Wang R, Yuan S, Geng W, Zhou W (2014). Quality of life after surgical resection compared with radiofrequency ablation for small hepatocellular carcinomas. Br J Surg.

[35] Salem R, Gilbertsen M, Butt Z, Memon K, Vouche M, Hickey R, Baker T, Abecassis M, Atassi R, Riaz A, Cella D, Burns J, Ganger D, Benson A, Mulcahy M, Kulik L, Lewandowski R (2013). Increased quality of life among hepatocellular carcinoma patients treated with radioembolization, compared with chemoembolization. Clin Gastroenterol Hepatol.

[36] Smits M, Pronk A, Nijsen F, Prince J, Zonnenberg B, van het Schip A, Lam M, van den Bosch M (2013). Quality of life in patients with hepatic malignancies treated with Holmium-166 radioembolization. J Vasc Interv Radiol.

