# Supplementary material for: Systematic review of patient reported quality of life following stereotactic ablative radiotherapy for primary and metastatic liver cancer
Source: Radiat Oncol. 2017 Jun 29;12:110. doi: 10.1186/s13014-017-0818-8 (PMC5492951; doi:10.1186/s13014-017-0818-8)
Supplement: Additional file 1: Appendix A. — Detailed search strategy. (DOCX 24 kb) [file 13014_2017_818_MOESM1_ESM.docx]

**Appendix A:**

Database: Ovid MEDLINE(R) without Revisions <1996 to March Week 2 2015> (with an updated search to October 2015)

Search Strategy:

--------------------------------------------------------------------------------

| 1 exp Stereotaxic Techniques/ (15415) |
| --- |
| 2 (stereotactic or stereotaxic).tw. (12648) |
| 3 (radiotherap: or radiosurg:).tw. (80988) |
| 4 2 and 3 (5732) |
| 5 exp Cranial Irradiation/ (2813) |
| 6 (cranial and radiosurg:).tw. (539) |
| 7 (extracranial and radiosurg:).tw. (317) |
| 8 exp Central Nervous System Neoplasms/sc [Secondary] (8943) |
| 9 (sbrt or sabr).tw. (1072) |
| 10 1 or 4 or 5 or 6 or 7 or 8 or 9 (26010) |
| 11 "Quality of Life"/ (108070) |
| 12 exp questionnaires/ (276773) |
| 13 karnofsky performance status/ (1697) |
| 14 karnofsky.tw. (3589) |
| 15 patient reported outcome:.tw. (3467) |
| 16 eastern cooperative oncology group.tw. (2252) |
| 17 ecog.tw. (3049) |
| 18 (16 or 17) and performance.tw. (2587) |
| 19 (euroqol 5d or eq 5d).tw. (3110) |
| 20 eortc qlq.tw. (1675) |
| 21 (short form 36 or sf 36).tw. (14897) |
| 22 functional assessment of cancer therapy.tw. (1091) |
| 23 (functional assessment of cancer therapy adj general).tw. (250) |
| 24 fact g.tw. (344) |
| 25 brief pain inventory.tw. (819) |
| 26 bpi.tw. (1082) |
| 27 11 or 12 or 13 or 13 or 14 or 15 or 18 or 19 or 20 or 21 or 22 or 23 or 24 or 25 or 26 (363720) |
| 28 10 and 27 (1287) |
| 29 (comment or letter or editorial or news or newspaper article or patient education handout or case reports or historical article).pt. (1930652) |
| 30 28 not 29 (1171) |
| 31 limit 30 to (english language and humans) (1066) |

Database: Embase <1996 to 2015 Week 11> (with an updated search to October 2015)

Search Strategy:

--------------------------------------------------------------------------------

| 1 exp stereotactic procedure/ (21508) |
| --- |
| 2 (cranial and radiosurg:).tw. (930) |
| 3 (extracranial and radiosurg:).tw. (547) |
| 4 exp brain tumor/ (84713) |
| 5 exp central nervous system tumor/ (160845) |
| 6 (stereotactic or stereotaxic or radiosurg:).tw. (26726) |
| 7 (4 or 5) and 6 (8001) |
| 8 (sbrt or sabr).tw. (3527) |
| 9 1 or 2 or 3 or 7 or 8 (25595) |
| 10 exp "quality of life"/ (276242) |
| 11 karnofsky performance status/ (4966) |
| 12 exp questionnaire/ (392465) |
| 13 patient reported outcome:.tw. (7812) |
| 14 eastern cooperative oncology group.tw. (3295) |
| 15 ecog.tw. (12516) |
| 16 (14 or 15) and performance.tw. (6868) |
| 17 (euroqol 5d or eq 5d).tw. (6464) |
| 18 eortc qlq.tw. (3572) |
| 19 (short form 36 or sf 36).tw. (23812) |
| 20 functional assessment of cancer therapy.tw. (1823) |
| 21 (functional assessment of cancer therapy adj general).tw. (395) |
| 22 fact g.tw. (739) |
| 23 brief pain inventory/ (1624) |
| 24 bpi.tw. (2114) |
| 25 10 or 11 or 12 or 13 or 16 or 17 or 18 or 19 or 20 or 21 or 22 or 23 or 24 (630443) |
| 26 9 and 25 (1818) |
| 27 (editorial or note or letter or erratum or short survey).pt. or letter/ or case study/ (1825117) |
| 28 26 not 27 (1664) |
| 29 limit 28 to (human and english language) (1464) |
